# Intelligent Robust Control Design with Closed-Loop Voltage Sensing for UPS Inverters in IoT Devices

**DOI:** 10.3390/s25133849

**Published:** 2025-06-20

**Authors:** En-Chih Chang, Yuan-Wei Tseng, Chun-An Cheng

**Affiliations:** Department of Electrical Engineering, I-Shou University, No. 1, Sec. 1, Syuecheng Rd., Dashu District, Kaohsiung City 84001, Taiwan; enchihchang@isu.edu.tw (E.-C.C.); yuanwei@isu.edu.tw (Y.-W.T.)

**Keywords:** voltage sensing, modified gray fast variable structure sliding mode control (MGFVSSMC), neural network (NN), UPS inverter, IoT device

## Abstract

High-performance UPS inverters prevent IoT devices from power outages, thus protecting critical data. This paper suggests an intelligent, robust control technique with closed-loop voltage sensing for UPS (uninterruptible power supply) inverters in IoT (internet of things) devices. The suggested control technique synthesizes a modified gray fast variable structure sliding mode control (MGFVSSMC) together with a neural network (NN). The MGFVSSMC allows system states to speedily converge towards the equilibrium within a shorter time while eliminating the problems of chattering and steady-state errors. The MGFVSSMC may experience state prediction errors when the UPS inverter is subjected to external highly nonlinear loads or internal parameters changing drastically. This results in high harmonic distortion and inferior dynamic response of the inverter output, affecting the guarding of the IoT device. An NN by means of a learning mechanism is employed to properly compensate for the prediction error of the MGFVSSMC, achieving a high-performance UPS inverter. The suggested control technique operates with one voltage sensing, which can yield fast transience and low inverter output-voltage distortion. Both simulations and digital signal processing (DSP) implementation results demonstrate the effectiveness of the suggested control technique under a variety of load conditions.

## 1. Introduction

Uninterruptible power supply (UPS) has been frequently used in internet of things (IoT)-based environments, such as street lighting systems, traffic management systems, and healthcare security systems [1,2,3,4]. An inverter serves as the core circuit in the UPS system, transforming direct current (DC) power into alternating current (AC) power. The UPS inverter will provide the backup power in the event of an unanticipated blackout so as to protect critical IoT devices and ensure system operation [5,6,7]. The design requisites of a high-performance UPS inverter usually depend on the following criteria: (1) voltage total harmonic distortion (THD) has to be low with respect to filter parameter variations or rectifier-type nonlinear loads; (2) fast transient behavior is desired when the load undergoes a dynamic change; and (3) steady-state error needs to be confined to the minimum possible level. Towards fulfilling the above-stated requirements, some control schemes have been reported in the literature, including repetitive controller, linear quadratic Gaussian (LQG) control, and mu-synthesis controller [8,9,10]. The repetitive controller and LQG control can well address perturbations and irregularities in system dynamics. However, they require accurate modeling of controlled plant dynamics while the control design and implementation are complicated. The mu-synthesis controller offers advantages in terms of system performance and robustness, but its control design may yield higher-order controllers. Variable structure sliding mode control (VSSMC) has gained widespread adoption due to its inherent insensitivity to both inner parametric alterations and outer load interferences [11,12,13]. The state of the system can be driven and held in a prescribed sliding surface. Within the motion of the trajectory governed by the sliding mode, the system state approximates near to the sliding surface. As soon as it contacts the sliding surface, the state behavior is converged in the direction of the origin following the sliding surface. Therefore, the system response has a strong suppression capability against interferences when sliding action occurs. The VSSMC with applications to UPS inverters has been presented in a wide range of literature. A four-leg inverter having a fixed switching frequency employs the SMC to regulate the output voltage suitable for UPS applications. This solution can substantially lessen the chattering phenomenon as well as its simplicity in realization. However, the dynamic behavior of the voltage drop recovering to the reference voltage under step load changing conditions is not sufficiently satisfactory [14]. There is an integral variable structure control applied to a UPS with the aim of following a sinusoidal voltage. The proposed scheme extended to a discrete version facilitates the digital implementation and introduces an integral compensator to suppress steady-state errors. The output voltage waveform still shows distortion under rectified loading, which implies that the chattering influence persists [15]. A discrete-time sliding mode controlled UPS inverter has been developed. A double loop control and smooth function are used in the presented controller. The chattering has been diminished; however, the weak control force leads to inferior transience during highly changing step loads [16]. The SMC utilizing differential equations with discontinuous right-hand sides is realized for UPS inverters. The described method is simple and easy to comprehend and yields low voltage THD at resistive loads. When a step change in load occurs, the voltage drop of the proposed UPS inverter cannot be quickly compensated because of the chattering problem [17]. With the foregoing classical VSSMCs, their state trajectories have a tendency to converge asymptotically in infinite time.

A type of fast variable structure sliding mode control (FVSSMC) is already being investigated in numerous works concerning its application [18,19,20,21,22]. It provides the ability to quickly accelerate the state trajectory to the equilibrium point throughout a finite period of time without singularities occurring. The FVSSMC may be practically subject to problems of chattering or steady-state errors. The reason is the difficulty in precisely estimating the fluctuations in system inner parameters as well as outer interferences. The chattering exists when the estimation of the system uncertainty boundary exceeds the limit, and vice versa, the steady-state error arises when the system uncertainty boundary becomes underestimated. Chattering is a high-frequency oscillation with the potential to provoke un-modeled dynamics, reduce control precision, and incur high heat loss in power electronics circuits. These lead to a situation where the sliding-mode presence and its robustness respecting interferences cannot be maintained further. Adaptive control methods and observers are usually utilized to estimate the system uncertainty boundaries when attempting to address the chattering [23,24,25]. The above-mentioned solutions can improve the quality of the transient or steady-state response. However, the control design is highly sophisticated with time-consuming operations and needs to be matched with exact system parameters. Gray prediction (GP) was proposed by Professor Tang in 1982, and afterwards it was successfully applied in diverse engineering fields [26,27,28]. It has been found to be effective in tackling uncertain systems forecasting problems. The GP relies on historic and existing information for analyzing and anticipating sequence variation tendency. Its main advantages come from computational simplicity and the small amount of data required for constructing the gray model (GM). It should be noted that there are a large number of approximate non-homogeneous data sequences existing in practical applications. The traditional GM fits the primitive data using the homogeneous exponential sequence, causing a forecasting imprecision. Thus, a modified gray model (MGM) with non-homogeneous exponential characteristics is adopted to obtain more exact forecasting results [29,30,31]. Through the MGM-compensated FVSSMC becoming MGFVSSMC, the chattering/steady-state error is surmounted if the uncertainty limit becomes exaggerated/underestimated. The MGFVSSMC may incur predictive errors during parametric sharp alterations and severely nonlinear loading occurrences. This creates a challenge regarding the output response of closed-loop UPS inverters. The neural network (NN) based on radial basis function (RBF) was introduced in the nineteen eighties [32,33,34]. It features fine ability for function approximation, often being used as a modeling of nonlinear functions [35,36,37,38]. Using an RBFNN with learning capability to compensate for MGFVSSMC’s prediction error becomes a good solution. As can be anticipated, the suggested control technique is capable of creating highly accurate trajectory tracking. For a variety of load cases, the presented UPS inverter can produce lower THD and quicker dynamic reaction. Results from simulations and experiments corroborate the effective performance of the suggested control technique.

## 2. Dynamic Modeling of the UPS Inverter

**Figure 1:**
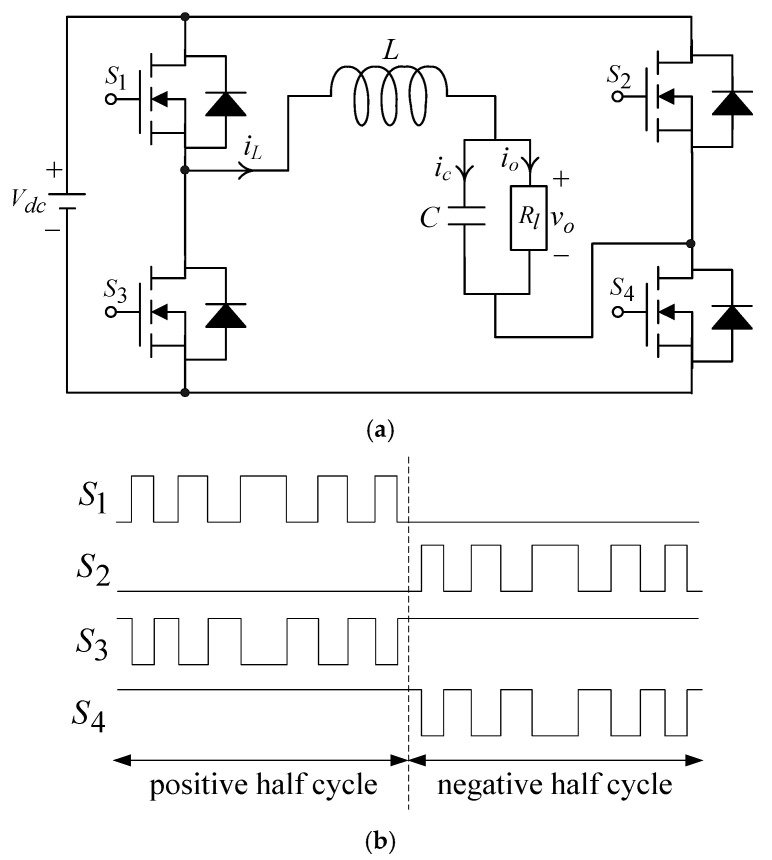
Configuration of a UPS inverter: (**a**) Circuit diagram. (**b**) Gate driving signal pattern.

The UPS inverter is illustrated in Figure 1a. It consists of four transistor switches, an output inductive–capacitive (LC) filter, and a resistive load ($R_{l}$). $V_{dc}$ denotes the DC link voltage; $i_{L}$ is the inductor current; $i_{c}$ represents the capacitor current; and $v_{o}$ and $i_{o}$ refer to the output voltage and current, respectively. Figure 1b depicts the gate drive signals ($S_{1}$, $S_{2}$, $S_{3}$, and $S_{4}$) obtained by comparing the sine wave reference signal of the demanded output frequency with the high-frequency triangular carrier signal. Consider the state variables $x_{1}=v_{o}$ and $x_{2}={\dot{x}}_{1}={\dot{v}}_{o}$, then the dynamical behavior n of the UPS inverter utilizing Kirchhoff’s voltage and current laws in Figure 1 becomes the following:

(1) $${\dot{x}}_{2}=-\frac{1}{LC}x_{1}-\frac{1}{R_{l}C}x_{2}+\frac{K_{inv}}{LC}u$$

where $K_{inv}$ represents the UPS inverter equivalence gain, and u stands for the control input.

The control design in a UPS inverter can be treated as a trajectory tracking issue. Let the demanded sine wave reference voltage be formulated as $v_{ref}=\sqrt{2}V_{rms}\sin(\omega t)$, where $V_{rms}$ and ω denote its root mean square value and angular frequency, respectively. This implies that the sine wave output voltage of the UPS inverter is necessary to follow a demanded AC reference voltage $v_{ref}$. The error state and its derivative can be denoted as $x_{e1}=x_{1}-v_{ref}$ and $x_{e2}={\dot{x}}_{e1}$, respectively. Taking (1) and the error state variables, the error dynamics of the UPS inverter produces the following:

(2) $${\dot{x}}_{e2}=a_{1}x_{e1}+a_{2}x_{e2}+bu+f$$

where $a_{1}=-1/LC$, $a_{2}=-1/R_{l}C$, $b=K_{inv}/LC$, and $f=-a_{1}v_{ref}-a_{2}{\dot{v}}_{ref}-{\ddot{v}}_{ref}$ symbolize uncertainties of the system. As shown in (2), a high-accuracy tracking error behavior can be achieved by well-designing control law u. This allows the state error $x_{e1}$ and its derivative $x_{e2}$ to converge to the equilibrium quickly during a limited time. In trajectory tracking control, the system dynamics behave as an insensitive stability while a quick closed-loop with restricted time convergence is created by the FVSSMC. A practical control of the FVSSMC designed with a UPS inverter provides quick dynamic recovery time and exact steady-state tracking. The chattering or steady-state error potentially occurs encircling the sliding surface due to system uncertain boundary overestimation or underestimation. An efficient MGM with non-homogeneous exponential behavior incorporating the FVSSMC is available to remove flutter or steady-state errors. Therefore, an MGFVSSMC is used to suppress chattering or steady-state errors. Under highly varied inner parameters or heavily nonlinear outer loads, the MGFVSSMC may yield modeling errors and unexpected perturbations. The predicted error then occurs, while the poor quality of the UPS inverter output voltage leads to a challenge in the protection of the IoT device. A radial basis function (RBF) neural network (NN) learns the predicted error of the MGFVSSMC, allowing the generation of high-performance UPS inverters. By synthesizing MGFVSSMC and NN, the intelligent, robust, closed-loop-controlled UPS inverter creates both immediate transient reaction and better steady-state behavior.

## 3. Control Design

Taking from the error dynamics (2), a variable structure sliding surface suitable for guaranteeing singularity-free quick time convergence to the equilibrium point is represented as follows:

(3) $$s=x_{e1}+\xi^{-1}x_{e2}^{\rho_{2}/\rho_{1}}$$

where ξ takes on a positive value, and $\rho_{1}$ and $\rho_{2}$ are positive odd numbers. In case the state error moves towards or away from the sliding surface, it is recommended to enhance the approach speed for quicker trajectory tracking. Thus, the sliding-mode approximation law can be specified as follows:

(4) $$\dot{s}=-\eta_{1}{\left|s\right|}^{\gamma_{1}}\tanh(\kappa s)-\eta_{2}s{\left|s\right|}^{\gamma_{2}sat(\left|s\right|-1)}-\eta_{3}s$$

where $\eta_{1}$, $\gamma_{1}$, κ, $\eta_{2}$, $\gamma_{2}$, and $\eta_{3}$ are all positive numbers, and sat(s)=s/δ denotes a smooth saturation function with a positive value δ.

The law u for the FVSSMC can be derived from (2)–(4) as follows:

(5) $$u=-b^{-1}[a_{1}x_{e1}+a_{2}x_{e2}+\xi \rho_{1}\rho_{2}^{-1}x_{e2}^{2-\rho_{2}/\rho_{1}}+\eta_{1}{\left|s\right|}^{\gamma_{1}}\tanh(\kappa s)+\eta_{2}s{\left|s\right|}^{\gamma_{2}sat(\left|s\right|-1)}+\eta_{3}s]$$

**Proof.**  With the aim of justifying the stability of the system at an equilibrium point, a Lyapunov candidate function V is expressed as follows:

(6) $$V=s^{2}/2$$

With the use of the error dynamics (2) and the control law (5), the differentiation of V with respect to time can be given as follows:

(7) $$\dot{V}=s\dot{s}=s{\left(x_{e1}+\xi^{-1}x_{e2}^{\rho_{2}/\rho_{1}}\right)}^{\prime }\leq -s\cdot [\eta_{1}{\left|s\right|}^{\gamma_{1}}\tanh(\kappa s)+\eta_{2}s{\left|s\right|}^{\gamma_{2}sat(\left|s\right|-1)}+\eta_{3}s]$$

□.

In fact, (7) reveals that both s and $x_{e2}$ will not be zero, allowing $\dot{V}$ to be less than zero. The s and its differential $\dot{s}$ can quickly converge to the equilibrium point for a limited time since (8) fulfills the Lyapunov’s stability criterion. The error trajectory of the system (2) also must be converged towards the equilibrium status quickly during a restricted time while using the control law (5). It should be taken into consideration that if the system uncertainty boundary becomes exaggerated or insufficiently estimated, chattering or steady-state error usually occurs. To overcome these concerns, an MGM with non-homogeneous exponential characteristics based on a centered approximation is employed. It samples no fewer than four output voltages to predict the state of the next output voltage. The operating instructions for the MGM are as follows:

Step 1: An n-item sequence containing non-negative values is expressed as follows:
  (8) $$\chi^{\left(0\right)}=\left\{x^{\left(0\right)}(1)\;,\;x^{\left(0\right)}(2),\;\cdot \cdot \cdot ,\;x^{\left(0\right)}(m)\right\}$$
Step 2: By allowing $\chi^{\left(1\right)}$ to be a first-order accumulated generated operation (1-AGO) sequence for $\chi^{\left(0\right)}$, one obtains the following:
  (9) $$\chi^{\left(1\right)}=\left\{x^{\left(1\right)}(1)\;,\;x^{\left(1\right)}(2),\;\cdot \cdot \cdot ,\;x^{\left(1\right)}(m)\right\}$$
  where $x^{\left(1\right)}(n)=\sum_{k=1}^{n}x^{\left(0\right)}(k)$, n=1, 2, ⋅⋅⋅, m.
Step 3: The first-order non-homogeneous differential gray model based on the $\chi^{\left(1\right)}$ can be built as follows:
  (10) $$\frac{dx^{\left(1\right)}}{dt}+\alpha x^{\left(1\right)}=\beta t$$
  where α denotes the developed coefficient of the model, and β stands for the gray action.

Let $Z^{\left(1\right)}$ be the adjacent neighbor mean-generating sequence of $\chi^{\left(1\right)}$, which becomes:

(11) $$Z^{\left(1\right)}=\left\{z^{\left(1\right)}(2),\;z^{\left(1\right)}(3),\;\ldots ,\;z^{\left(1\right)}(m)\right\}$$

The background value $z^{\left(1\right)}(n)$ of the gray model is then expressed as follows:

(12) $$z^{\left(1\right)}(n)=\frac{1}{2}(x^{\left(1\right)}(n)+x^{\left(1\right)}(n-1))$$

where n=2, 3, ⋅⋅⋅, m.

The discrete sequence of (9) can be represented as follows:

(13) $$x^{\left(0\right)}(n)+\alpha z^{\left(1\right)}(n)=\beta$$

For determination of α and β values, (13) can be formulated as follows:

(14) Y=Ψζ

where $Y=\left[\begin{array}{c} x^{\left(0\right)}(2)\\ x^{\left(0\right)}(3)\\ \cdot \\ \begin{array}{l} \cdot \\ \cdot \end{array}\\ x^{\left(0\right)}(m) \end{array}\right]$, $\Psi =\left[\begin{array}{cc} -\frac{1}{2}[x^{\left(1\right)}(1)+x^{\left(1\right)}(2)] & 1\\ -\frac{1}{2}[x^{\left(1\right)}(2)+x^{\left(1\right)}(3)] & 1\\ \cdot & \cdot \\ \begin{array}{l} \cdot \\ \cdot \end{array} & \begin{array}{l} \cdot \\ \cdot \end{array}\\ -\frac{1}{2}[x^{\left(1\right)}(m-1)+x^{\left(1\right)}(m)] & 1 \end{array}\right]\;$, and $\zeta =\left[\begin{array}{c} \alpha \\ \beta \end{array}\right]$.

Both α and β can be solved by the least squares method as follows:

(15) $$\zeta =\left[\begin{array}{c} \alpha \\ \beta \end{array}\right]={\left(\Psi^{T}\Psi \right)}^{-1}\Psi^{T}Y$$

By substituting the estimation parameters α and β into (10), the function of the time response is as follows:

(16) $${\hat{x}}^{\left(1\right)}(n)=(x^{\left(0\right)}(1)+\frac{\beta }{\alpha^{2}}-\frac{\beta }{\alpha })e^{-\alpha (n-1)}+\frac{\beta }{\alpha }n-\frac{\beta }{\alpha^{2}}$$

Step 4: With the inverse AGO, the predicted expression for the primitive sequence yields the following:
  (17) $${\hat{x}}^{\left(0\right)}(n+1)={\hat{x}}^{\left(1\right)}(n+1)-{\hat{x}}^{\left(1\right)}(n)=(x^{\left(0\right)}(1)+\frac{\beta }{\alpha^{2}}-\frac{\beta }{\alpha })(1-e^{\alpha })e^{-\alpha n}+\frac{\beta }{\alpha }$$

**Figure 2:**
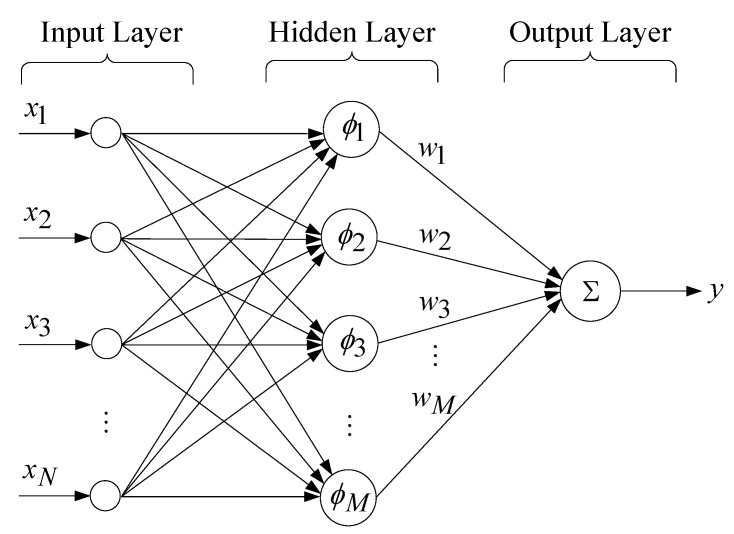
Architecture of an RBFNN.

In the final step, the control law in (5) is augmented with an MGM term ($u_{mgm}(n)$) for the suppression of chattering:

(18) $$u_{mgm}(n)=\left\{\begin{array}{c} 0\\ \Gamma \hat{s}(n)sat(s(n)\hat{s}(n)) \end{array}\begin{array}{c} ,\;\left|\hat{s}(n)\right|<\varepsilon \\ ,\;\left|\hat{s}(n)\right|\geq \varepsilon \end{array}\right.$$

where Γ represents a constant, $\hat{s}(n)$ is a forecast of s(n), sat(⋅) denotes a saturated continuous function, and ε symbolizes the system boundary. When the load on the UPS inverter becomes strictly nonlinear, the MGFVSSMC becomes subject to inaccurate prediction errors. The RBFNN is exploited to ameliorate such an issue in order to elevate the system’s performance. The architecture of RBFNN is depicted in Figure 2. It consists of an input layer, a hidden layer, and an output layer.

The input layer to the hidden layer represents a nonlinear transformation, which is performed by the RBF. Let the input of the RBFNN be as follows:

(19) $$x={\left[x_{1},\;x_{2},\;\ldots ,\;x_{N}\right]}^{T}$$

A Gaussian function selected as the radial basis function $\phi_{j}$ of the RBFNN can be formulated as follows:

(20) $$\phi_{j}=\exp(-\frac{{\left\|x-c_{j}\right\|}^{2}}{2r_{j}^{2}}),j=1,\;2,\;\ldots ,\;M$$

where $c_{j}$ refers to the central vector corresponding to the *j*-th hidden layer neuron, $\left\|\cdot \right\|$ stands for Euclidean distance, and $r_{j}$ denotes the width vector of the Gaussian function. The number of hidden neurons, the structure of the input vectors, the choice of Gaussian width, and the design of the center for the basis functions deserve to be further explained below. When a large number of hidden neurons are served in the network, it will result in a higher performance of the NN. With needlessly more neurons, there is a potential for overfitting. When the number of hidden neurons becomes insufficient to recognize the messages from a complicated dataset, underfitting may happen. The number of hidden neurons can equal the number of training patterns. However, the empirical rule suggested that the number of hidden neurons is usually much smaller than the number of input layer neurons [39,40]. The structure of input vectors with N-dimensional data points comes from the sampling of the MGM output in (18). The Gaussian width $r_{j}$ governs the interpolation function’s smoothing. As shown in (21), the nearest neighbor method is recommended to derive the width $r_{j}$ [41,42]:

(21) $$r_{j}=\eta \times \min(\left\|c_{j}-c_{i}\right\|)$$

where η is a positive overlapping constant, and $c_{i}$ denotes the clustering center vector.

The center $c_{j}$ is designed to perform a comparison of network input vectors for yielding a response with radial symmetry. The K-means clustering algorithm helps to search a set of centers, giving a better representation of the spread of data points. It preselects the number of centers K, and partitions the data points $x^{p}$ into K unlinked subsets $\Omega_{j}$ and $N_{j}$ data points. The $c_{j}$ represents the center of data points from the set $\Omega_{j}$, as given by [43,44]:

(22) $$c_{j}=\frac{1}{N_{j}}\sum_{p\in \Omega_{j}}x^{p}$$

**Figure 3:**
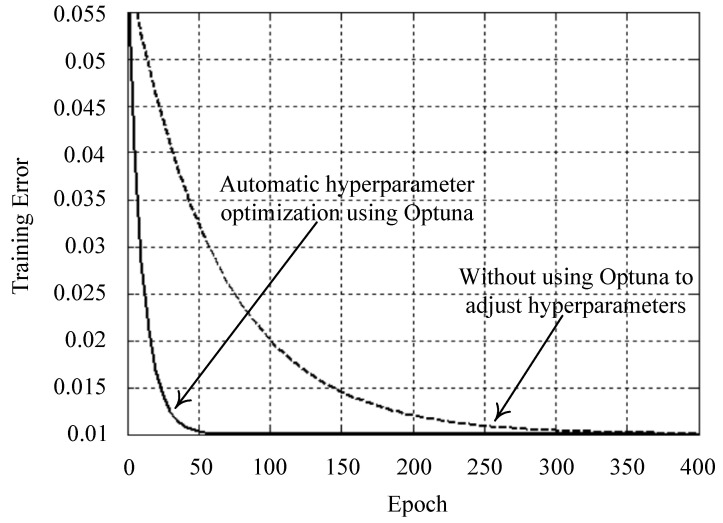
Comparison of training errors.

The relationship between the hidden layer and the output layer consists of a linear transformation. The respective outputs from each hidden node ${\left[\phi_{1},\;\phi_{2},\;\ldots ,\;\phi_{M}\right]}^{T}$ are multiplied by the weights ${\left[w_{1},\;w_{2},\;\ldots ,\;w_{M}\right]}^{T}$ for generating the output response of the network as follows:

(23) $$y=\sum_{j=1}^{M}w_{j}\phi_{j}$$

where the weights $w_{j}$ can be calculated by the pseudo-inverse method [45,46]. With the aforementioned transformation relations, the RBFNN can approximate the nonlinear function that achieves the learning ability. Specifically, the gray forecast output of the (17) is initially assigned as the NN input data in the input layer. Then, the processing inputs are applied through Gaussian RBF, mapping them into the output layer. The final output function at the output layer allows for more accurate prediction results. The pseudocode of the whole method process is shown in Algorithm 1. To assist researchers in employing fewer resources and reducing training time, an Optuna framework is adopted to optimize the hyperparameters of the model [47,48]. This automatic hyperparameter optimization software promotes the predictive precision of the model [49,50]. Figure 3 compares the training error using Optuna and without Optuna for adjusting the hyperparameters. Using Optuna to optimize hyperparameters produces smaller training errors with fewer epochs.
**Algorithm 1.** Pseudocode of the whole method Define all system parameters based on (1). Initialize all width, center, and weight vectors in the RBFNN. For k = 1:N Define the reference sine wave. Compute the measured output sine wave from (1). Compute the error of the state. Choose the sliding surface as given in (3) and the sliding-mode approximation rule as described in (4). Compute the control law from the FVSSMC with (5). Sample the measured output sine wave. Execute MGM steps 1 to 4 to predict output by using (8) to (17). Input predict sample data using (17) into input layer of RBFNN. Compute the width and center from (21) and (22). Update weight vectors to get RBFNN output from (23). End for

**Figure 4:**
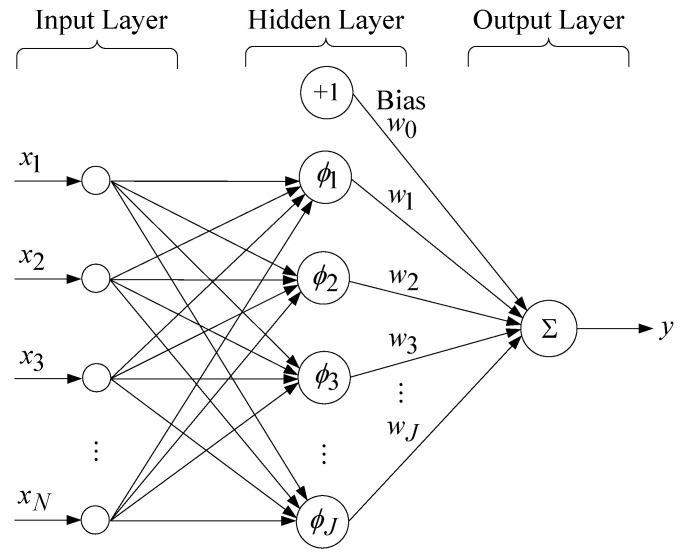
Architecture of a generalized RBFNN.

One should be aware that the complexity of training the RBFNN relies on the training elements N, and the hidden neurons M. The complexity normally follows an O(N×M) increment. To reduce the difficulty in calculation and the network complexity, a generalized RBFNN has been considered, as illustrated in Figure 4 [51]. This model generalization is performed using the Galerkin scheme. The number of hidden layer nodes is J (often less than the number of training examples N). One of the weights in the output layer is assigned as a bias (i.e., a variable unrelated to the data), and the related RBF is treated as a constant of positive one. While the number of training elements grows, the real-time computational load becomes higher as well. The optimal number of hidden neurons and the effective training algorithms can handle the computational load. Cross-validation can be used to decide the optimal number of hidden neurons and discover how to stop training at the best time [52].

## 4. Simulation and Experimental Results

The Matlab (version 6.1)/Simulink (version 4.1) package has been employed to simulate the UPS inverter using the suggested control technique and the classical SMC. Table 1 displays the system parameters of the UPS inverter.

**Figure 5:**
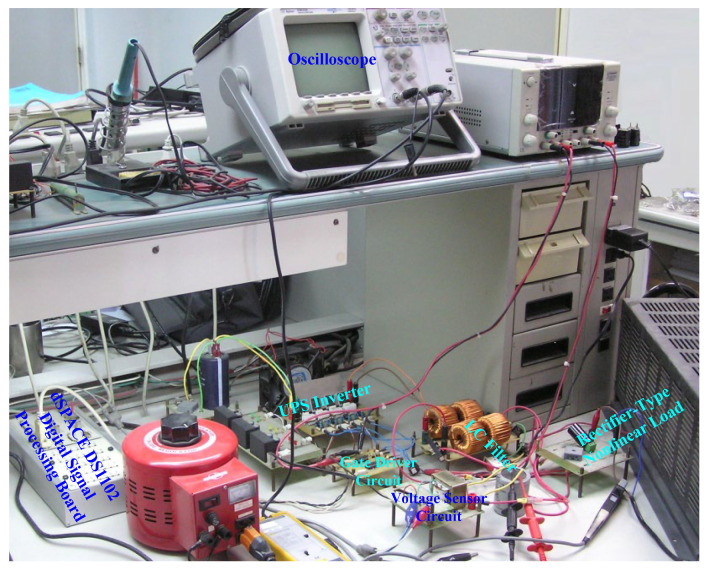
Photo of physical equipment and experimental environment.

The UPS inverter is implemented on a dSPACE DS1102 (dSPACE GmbH, Paderborn, Germany) control board based on a TMS320C31 (Texas Instruments, Dallas, TX, USA), 32-bit floating-point digital signal processor. It includes 128 K words of memory, which provides enough speed to permit zero-wait-state execution. The efficient performance of dSPACE DS1102 (dSPACE GmbH, Paderborn, Germany) allows the TMS320C31 (Texas Instruments, Dallas, TX, USA) to run at 40 MHz with a 50 ns single-cycle instruction execution time. The suggested algorithm is automatically transformed as real-time C code by the Matlab/Simulink for implementation on the dSPACE. With four optocouplers (PC923 (Sharp Corporation, Sakai City, Osaka, Japan)), the control and power signals can be insulated separately. The IRF460 is adopted for the power MOSFET. The voltage sensor is based on the AD202 (Analog Devices Inc., Wilmington, MA, USA) isolated amplifier. Figure 5 shows the photo of physical equipment and the experimental environment.

**Figure 6:**
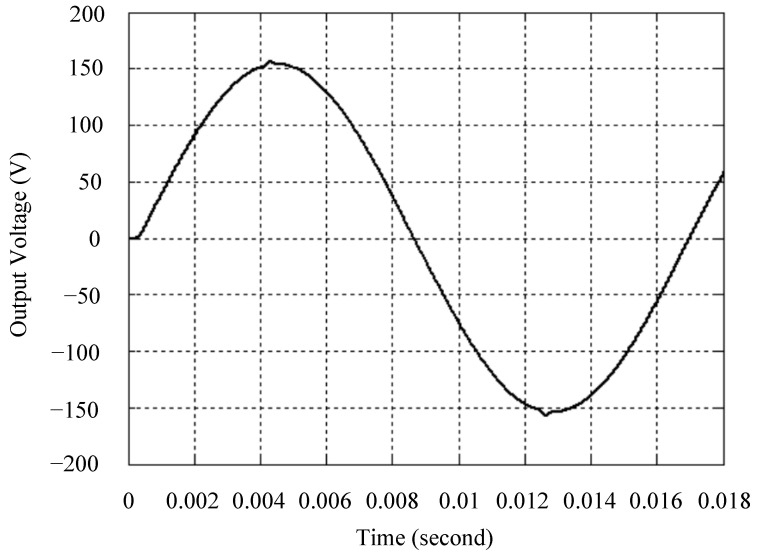
Simulated output voltage for the suggested control technique under TRIAC-controlled load with abrupt loading removal.

**Figure 7:**
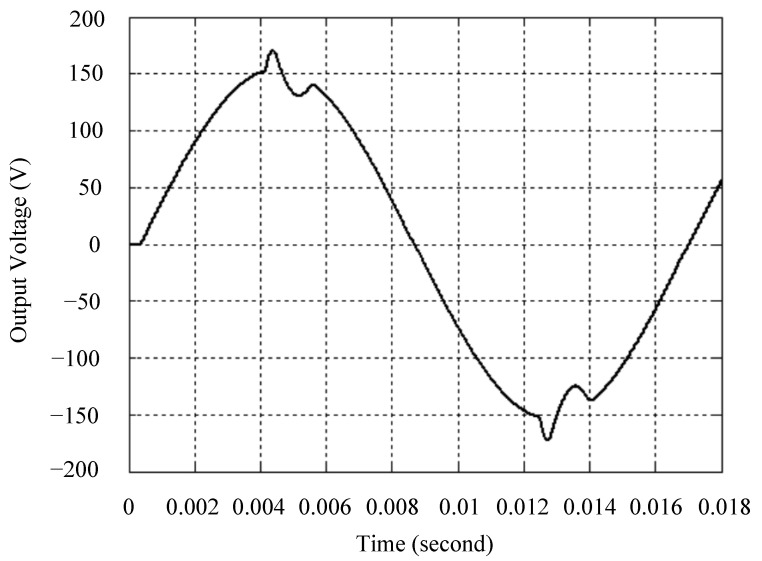
Simulated output voltage for the classical SMC under TRIAC-controlled load with abrupt loading removal.

**Figure 8:**
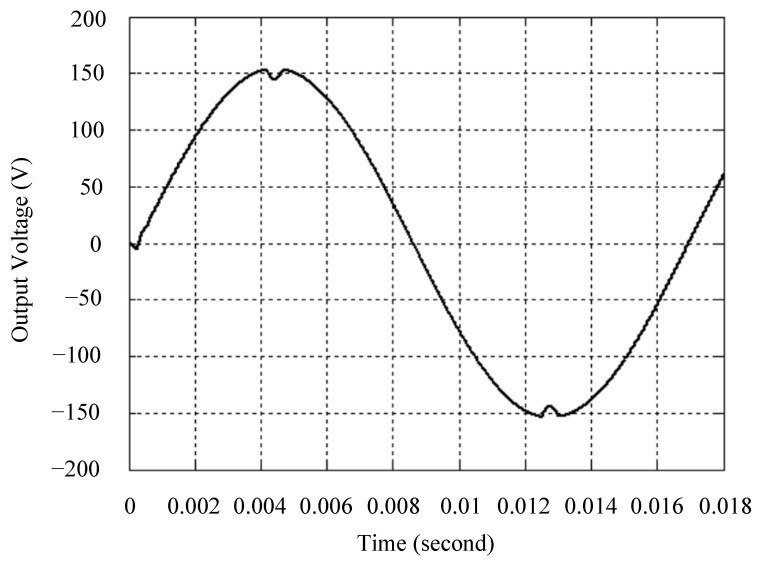
Simulated output voltage for the suggested control technique under TRIAC-controlled load with abrupt loading increase.

**Figure 9:**
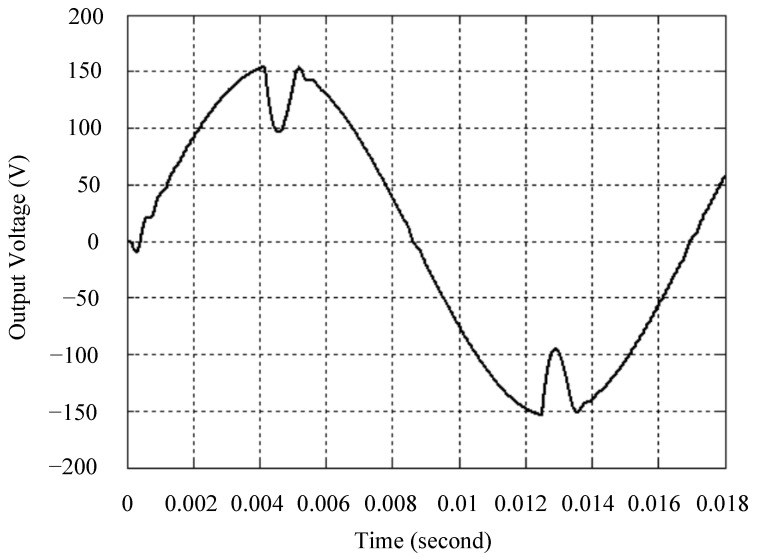
Simulated output voltage for the classical SMC under TRIAC-controlled load with abrupt loading increase.

**Figure 10:**
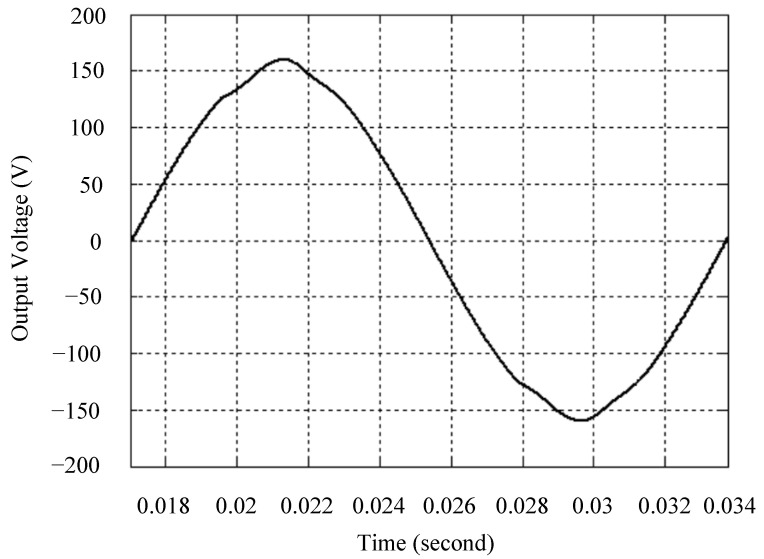
Simulated output voltage for the suggested control technique under rectifier-type nonlinear loading.

**Figure 11:**
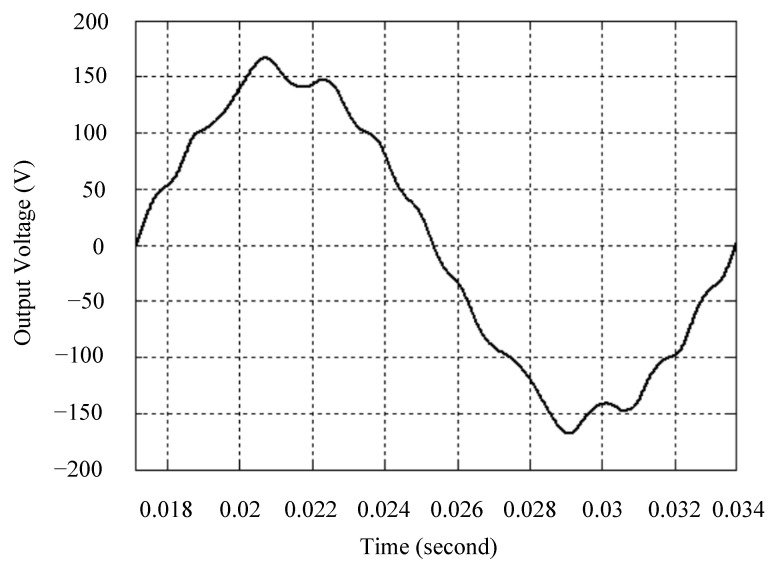
Simulated output voltage for the classical SMC under rectifier-type nonlinear loading.

**Figure 12:**
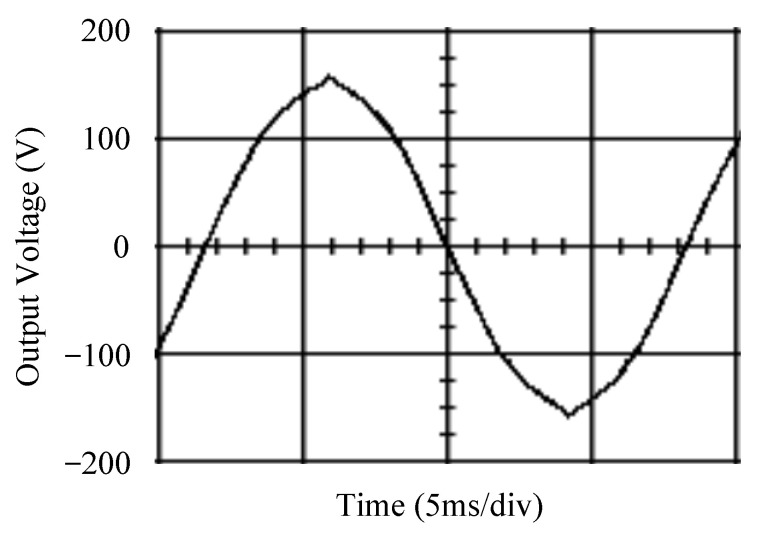
Experimental output voltage for the suggested control technique under TRIAC-controlled load with abrupt loading removal.

**Figure 13:**
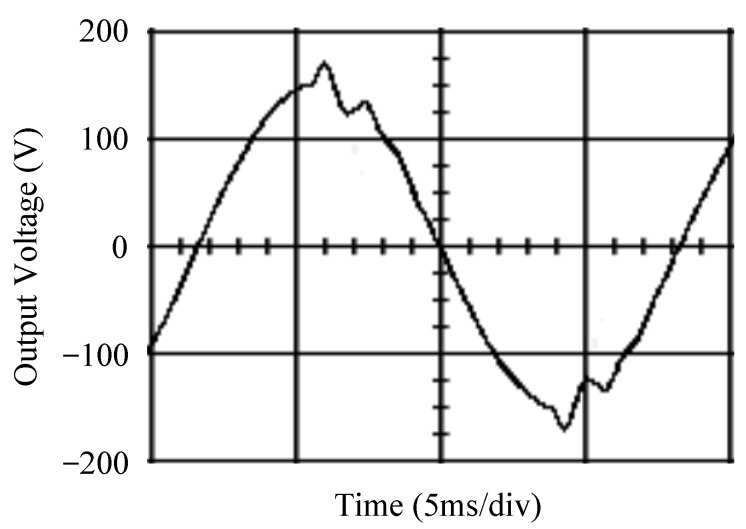
Experimental output voltage for the classical SMC under TRIAC-controlled load with abrupt loading removal.

**Figure 14:**
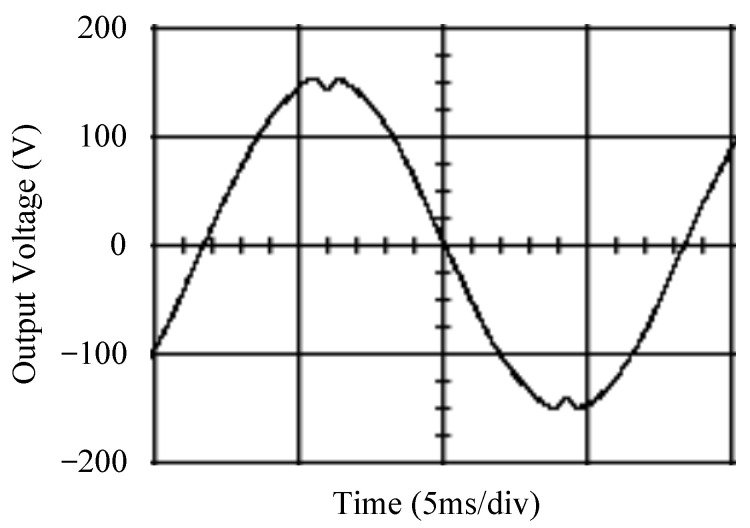
Experimental output voltage for the suggested control technique under TRIAC-controlled load with abrupt loading increase.

**Figure 15:**
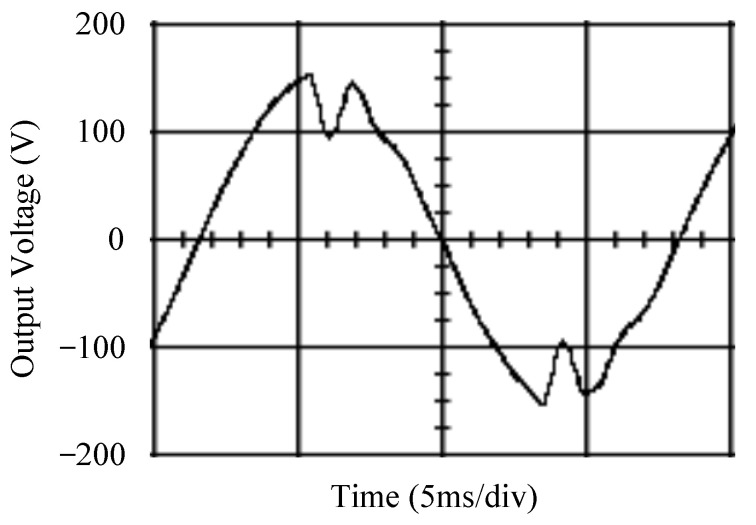
Experimental output voltage for the classical SMC under TRIAC-controlled load with abrupt loading increase.

**Figure 16:**
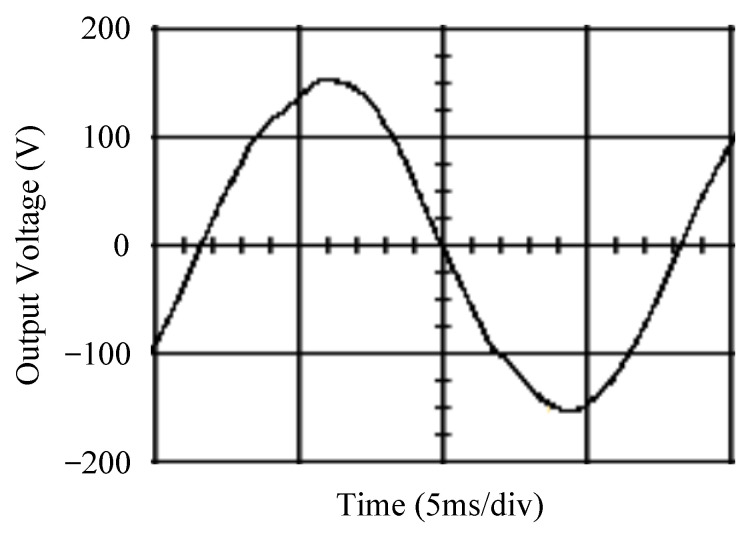
Experimental output voltage for the suggested control technique under rectifier-type nonlinear loading.

**Figure 17:**
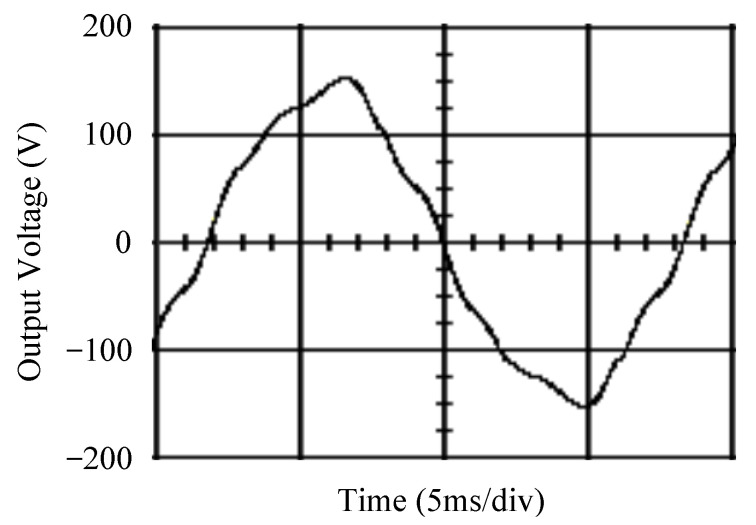
Experimental output voltage for the classical SMC under rectifier-type nonlinear loading.

**Figure 18:**
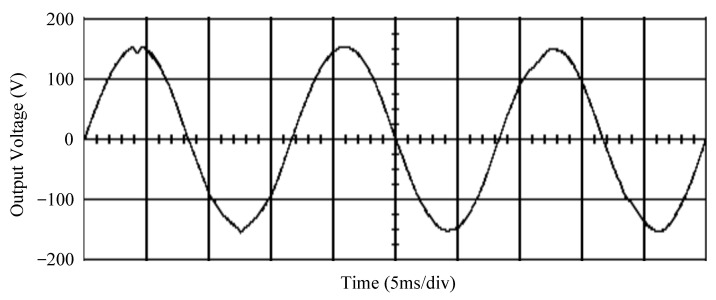
Experimental output voltage for the suggested control technique under a wider operating range with dynamic loading.

**Figure 19:**
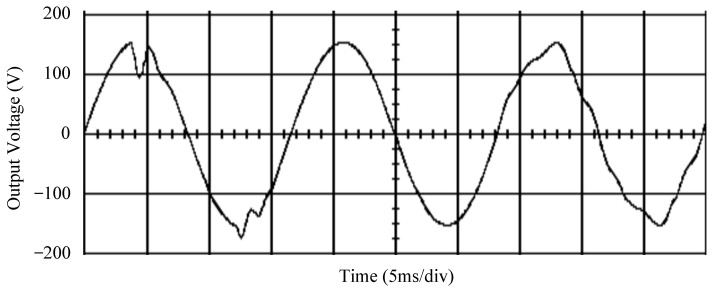
Experimental output voltage for the classical SMC under a wider operating range with dynamic loading.

The simulated output voltage waveforms of the UPS inverter with the suggested control technique and the classical SMC are depicted in Figure 6 and Figure 7, respectively. They are tested under TRIAC (triode for alternating current)-controlled loading at the 90/270 firing angles from full load to no load. The proposed strategy demonstrates a slight voltage swell, which can be regarded as approximately corresponding to the referred voltage waveform. The classical SMC has a higher voltage swell of about ten percent over a nominal referred value. The voltage swell of the suggested inverter is equal to 1.26 volts, while the voltage swell of the classical SMC inverter is 15.21 volts. The ratio of voltage swell between the two methods is approximately 12 times, which reveals the weak transient recovery ability of the classical SMC. Figure 8 and Figure 9, respectively, indicate the simulated output voltage waveforms of the UPS inverter using the suggested control technique and the classical SMC. The two methods are verified under TRIAC-controlled loading at the 90/270 firing angle from no load to full load. The suggested control technique establishes a quite minor instantaneous voltage dip followed by a speedy return to the reference voltage. In contrast, there is a large instantaneous voltage dip for the classical SMC, showing limited ability in the transient recovery to the steady state. There is a voltage dip of 10.78 volts in the suggested inverter, as compared to 59.42 volts in the classical SMC inverter. The ability of the suggested control technique in restraining voltage dip is about 5.5 times that of the classical SMC. This implies that the suggested control technique with its rapidly dynamic behavior can compensate for the voltage dip well. The simulated output voltage waveforms of the UPS inverter for the suggested control technique and the classical SMC at rectifier-type nonlinear loading are displayed in Figure 10 and Figure 11, respectively. The suggested control technique results in a low THD for the UPS inverter output voltage, exhibiting a robust AC steady-state response. The classical SMC causes the UPS inverter output voltage to have significant distortion, which appears as a non-sinusoidal waveform of high THD. The voltage THD percentage of the suggested inverter is 0.61%, whereas the classical SMC inverter has a voltage THD of 18.79%. The classical SMC has unsatisfactory THD that is nearly four times greater than the industry standard. Figure 12 and Figure 13, respectively, represent the experimental output voltage waveforms of the proposed UPS inverter and the classical SMC UPS inverter. These are checked under TRIAC-controlled load at 90 and 270 degrees firing angles with abrupt load removal. The suggested control technique is capable of producing little voltage swell along with a very short transient recovery time at the firing angle. The classical SMC shows a large voltage swell with weak transient compensation and even fails to meet industrial standard requirements. The voltage swell of the suggested inverter varies slightly by 2.47 volts at the quarter cycle. The voltage swell of the classical inverter severely expands to 16.32 volts at the three-fourths cycle. During a large voltage swell, the insulation of sensitive devices is subjected to greater stress, causing device failures. The experimental UPS inverter output voltage waveform is illustrated in Figure 14 using the suggested control technique for TRIAC-controlled loading at 90 and 270 degrees firing angles with abrupt load increase. The suggested control technique establishes a very slight voltage dip and then rapidly goes back to the reference voltage level. Figure 15 presents the experimental UPS inverter output voltage waveform obtained from the classical SMC operating under the same loading situation. The classical SMC obviously increases the output voltage dip of the UPS inverter, leading to an undesirable dynamic behavior during returning to the sine wave reference. During the positive half cycle, the suggested inverter shows a minor voltage dip of 11.82 volts, while the classical SMC inverter exhibits a severe voltage dip of 61.81 volts during the negative half cycle. A large instantaneous voltage dip imposes a burden on the inverter, which may shut down the device or induce a malfunction. The experimental output voltage waveform of the UPS inverter with the suggested control technique at rectifier-type nonlinear loading is displayed in Figure 16. The output voltage has a low THD value, well approximated to the sine reference voltage, exhibiting a fine, robust insensitivity. The experimental output voltage waveform of the UPS inverter with the classical SMC at rectifier-type nonlinear loading is given in Figure 17. A high output voltage THD reveals heavy vibration and distortion waveform. The suggested inverter offers a voltage THD percentage of 0.59%, as opposed to the 19.14% for the classic SMC inverter. The high harmonic causes waveform deformation, which makes the protection device operate incorrectly. Sometimes it even leads to overheating and shortens the life of the device. The simulated and experimental results comparing the suggested control technique with the classical SMC are summarized in Table 2 and Table 3, respectively. Figure 18 reveals the experimental UPS inverter output voltage waveform for the suggested control technique under a wider operating range of dynamic loading. In the first cycle, the inverter experiences a sudden load increase in the positive half cycle and a sudden load removal in the negative half cycle. During the second cycle, the inverter works at a full load. In the last cycle, a rectifier-type nonlinear load is applied to the inverter. From the beginning cycle until the final cycle, the suggested control technique establishes a fast dynamic response and robust insensitivity against interferences. The experimental UPS inverter output voltage waveform for the classical SMC under the same test condition is presented in Figure 19. The inverter operates under a linear resistive load during the second cycle, so the waveform appears almost sine wave. During other cycles, the output voltage drops and rises greatly and suffers from severe harmonic distortion, exceeding industry standards. The suggested control technique has provided fewer voltage swells and sags and lower THD throughout various load testing cases. These yield shorter convergence time and higher tracking precision of the steady-state behavior. With respect to the performance expectations of international standards, both the IEEE (Institute of Electrical and Electronics Engineers) Standard 519-2014 [53] and the European Standard EN50160:2010 [54] suggested practice restrict low-voltage systems below one kilovolt with a voltage THD tolerance of eight percent. In the case of medium-voltage systems ranging from one to sixty-nine kilovolts, the IEEE Standard 519-2014 and the European Standard EN50160:2010 advise the voltage THD restriction of five percent and eight percent, respectively. The voltage THD of the suggested converter is far below eight percent when compared to both the IEEE Standard 519-2014 and the European Standard EN50160:2010. Regarding voltage drop and swell, IEEE Standard 1159-2019 [55] restricts the voltage drop to the range of 0.1 to 0.9 per unit. The measured value represents the root mean square (RMS) of the voltage for the power supply frequency within one-half cycle to one minute. The voltage swell is specified as an incremental increase in voltage RMS exceeding 1.1 per unit with a duration of one-half cycle to one minute. Based on the definition of the European Standard EN50160:2010, the voltage drop occurs when the RMS voltage at a certain point from the electrical system decreases momentarily to a prescribed starting critical level, typically 90% of the reference voltage, with a duration of between one-half cycle and one minute. The voltage increase starting threshold corresponds to 110% of the reference voltage with the duration of ten milliseconds to one minute. By using the suggested control technique, the voltage dip and swell caused by the UPS inverter satisfy the requirements of the IEEE Standard 519-2014 and the European Standard EN50160:2010. This implies that the suggested UPS inverter establishes a fast transient response during transient loading before reverting back to the reference sine wave. In addition, the THD, overshoot, and settling time are investigated. These are compared with the values reported in recent related studies to highlight the performance of the proposed system. A continuous SMC is presented to control the voltage source inverter. The saturated function based on the limiting boundary is used to mitigate the chattering. A high-gain state observer is also employed to evaluate the inductor current during loading interferences. The result reveals a THD of 1.14% under nonlinear loading [56]. The suggested control technique proposed in this paper can obtain a THD of less than 0.7% at the same load. A dynamic sliding mode controlled single-stage boost inverter is developed [57]. This method utilizes a single loop associated with a modified sliding surface to address the system uncertainty. There is a low THD of 0.91% under varying load and nearly no overshoot. In the paper, the suggested method achieves a very low THD of 0.59% at heavily nonlinear loading, as well as being virtually free of overshoots. The strategy incorporating PI control and SMC has been practiced in grid-tied inverters for suppressing the THD [58]. It can operate at a fixed switching frequency to adapt to the variation of the grid environment. The THD approaches 5%, and the settling time is 3.13 ms, which is acceptable performance. Under load fluctuations, the THD of the suggested control technique proposed in this paper is far less than 5%, and the settling time reaches 1.45 ms. As analyzed in the above-mentioned summary, the methodology presented in this paper can be regarded as advantageous over recent works. The steady state and transient responses have emphasized the contribution and practical impact. Notably, the proposed UPS inverter using a single output voltage sensor has yielded good dynamic and steady-state response. In future research efforts, it can be considered to employ UPS inverters operating in parallel as well as additional current sensors. The stability and fault tolerance of the system can be strengthened. Although such a structure increases circuit complexity, it provides redundancy, reinforces dynamic response, and yields better steady-state accuracy.

## 5. Discussion

Both simulated and experimental findings of the suggested UPS inverter comply with the performance requisites of IEEE Standard 519-2014 and the European Standard EN50160:2010 for THD, voltage dip, and swell. The design of the output LC filter in the inverter represents an importance regarding the performance. It can be designed from the recommendations of earlier reference works [59,60,61]. With practical application considerations, the size of the filters should preferably be decreased. This needs to be matched by a relatively high switching frequency, normally between 20 kHz and 40 kHz, depending on metal-oxide semiconductor field-effect transistors. A factor correlated to the cutoff frequency of the output LC filter needs to be taken into account. Decreasing the value of this factor will result in a great decrement and minor amplification of the switching frequency and the fundamental frequency, respectively. If this factor value were to be minimized, the modulation value should be ideally designed at 0.95 or below. It is necessary to determine a critical factor in relation to the switching frequency and the inductor ripple current. Its value prefers to be in the limit of twenty percent to forty percent. Based on the aforementioned considerations, the element value of the output LC filter can be estimated.

The other issue of attention is to increase greater robustness of closed-loop control systems with different types of sliding mode controls, like complex-valued sliding mode control (CVSMC) and double-integral sliding mode control (DISMC). The CVSMC can retrieve the lost dynamics as well as diminish the chattering, while the DISMC can reduce the steady-state error and improve the transient response. A complex-valued sliding plane is proposed to control and observe induction motors. It addresses the control problem of original coordinates by not requiring an extra frame of reference. In spite of its heavily nonlinear sliding manifolds, this method streamlines the composition and analysis of the algorithms [62]. In order to synchronize the doubly fed induction generator with both imbalanced and distorted harmonic power grids, the CVSMC algorithm is introduced. Such an algorithm is chattering-free, and system state limited-time convergence is justified in the context of parameter variations and perturbations [63]. A sliding mode control designed with complex values has been introduced in the application of electric machinery. The arrival times of the CVSMC and the classical SMC are comparatively analyzed under the same preliminary circumstance and control force. The findings reveal that the complex number-based sliding mode design yields a faster arriving time towards the sliding manifold than the classical sliding mode design [64]. There is a DISMC presented to overcome the problem of asymmetrical and harmonic currents in a dual three-phase permanent magnet synchronous machine. It offers simplicity of construction as well as the absence of coordination transformations. This proposed approach can mitigate the vibration of the current during transient causes, while the steady-state error is avoided due to the integrator effect [65]. To control a pulse width-modulated DC-DC buck converter, a simplified DISMC methodology has been designed. The control law is deduced from the equivalent control, allowing for the suppression of greater interferences. The proposed solution is algorithmically unsophisticated with low realization cost and can establish quick transience and robust tracking behavior [66]. The DISMC based on pulse width modulation is examined to accomplish a converter’s voltage regulation in case of input voltage and load interferences. The presented algorithm is implemented using a field-programmable gate array for controlling the DC-DC boost converter. The proposed converter features quick dynamic response, decreased starting transient, and steady-state voltage regulation among different perturbations [67]. Additionally, some recent papers in the state-of-the-art considerations are provided for readers’ reference. These represent the application of integrating VSSMC, GP, and NN to enhance system performance [68,69,70,71].

## 6. Conclusions

In this paper, a modified gray model compensated FVSSMC together with an NN learning mechanism can control the UPS inverter suitable for the use of IoT devices. The system trajectory of a classical SMC into sliding motion can be impervious against both parametric fluctuations and loading interferences. One should be aware that a long time convergence to the equilibrium point occurs. Such a slow convergence makes the tracking trajectory susceptible to uncertain perturbations, weakening system robustness. The FVSSMC provides no singularity property and also accelerates the system state to converge towards the origin within a restricted time. When the system uncertainty bound is exaggerated or underestimated, the chattering or steady-state error may arise. The chattering increases the UPS inverter’s energy loss and introduces high-frequency harmonics into its output voltage. The steady-state error decreases the control precision, which results in the system state being hard to attain the desired sine wave. Since FVSSMC combines the MGM compensation as MGFVSSMC, the chattering and steady-state errors are addressed. However, the prediction error may emerge in the MGFVSSMC under highly nonlinear loading. The RBFNN is employed to learn the MGFVSSMC’s prediction error with the aim of obtaining a precisely predicted output. The proposed control technique leads to a high-performance, feedback-controlled UPS inverter. Whether under TRIAC-controlled loads or heavily nonlinear loads, the output voltage of the proposed UPS inverter displays rapid transience and low harmonic distortion. Both computer simulation results and digital signal processing (DSP)-based experimental results demonstrate the feasibility of the proposed control technique. Although MGM provides better predictions than traditional GM, a drawback still exists. The MGM utilizes a transformation from difference to differential equations for solving the time response formula. This will result in a transformation error, affecting the accuracy of the prediction. A type of non-homogeneous exponential discrete gray model can be recommended to enhance the MGM performance [72,73]. Also, the authors believe that the proposed control technique can maintain low THD and fast transient response during long-term operation (as shown in Figure 18 and Figure 19). To achieve higher performance stability and better protection against environmental disturbances (e.g., electromagnetic interference (EMI)), the use of inverters operating in parallel and EMI filters can be considered. The parallel UPS inverter with dual-loop control structure can enhance the dynamic response, stability, and redundancy [74]. The strong EMI may degrade UPS inverter performance. The EMI filters can be designed into the UPS inverter with less adverse impact on performance [75,76]. The dSPACE DSP platform provides the advantage of allowing rapid prototype development of the controller. To reduce development costs, the proposed control architecture is also suitable for integration into commercial DSP platforms. For example, the use of a field programmable gate array (FPGA) to realize the proposed control architecture provides both commercial and cost advantages [77]. These are all potential future research directions.

## Figures and Tables

**Table 1:** System parameters of the UPS inverter.

Parameters	Values
DC-link voltage ($V_{dc}$)	200 V
Filter inductor (L)	1 mH
Filter capacitor (C)	20 μF
Resistive load ($R_{l}$)	12 ohm
Switching frequency	30 kHz
AC output voltage ($v_{o}$)	$110\sqrt{2}$ V
Frequency of AC output voltage	60 Hz

**Table 2:** Simulated voltage dip, swell, and THD.

Methods	Results		
Simulations (Suggested control technique)	Abrupt loading removal	Abrupt loading increase	Rectifier-type nonlinear loading
	Voltage swell	Voltage dip	THD
	1.26 V	10.78 V	0.61%
Simulations (Classical SMC)	Abrupt loading removal	Abrupt loading increase	Rectifier-type nonlinear loading
	Voltage swell	Voltage dip	THD
	15.21 V	59.42 V	18.73%

**Table 3:** Experimental voltage dip, swell, and THD.

Methods	Results		
Experiments (Suggested control technique)	Abrupt loading removal	Abrupt loading increase	Rectifier-type nonlinear loading
	Voltage swell	Voltage dip	THD
	2.47 V	11.82 V	0.59%
Experiments (Classical SMC)	Abrupt loading removal	Abrupt loading increase	Rectifier-type nonlinear loading
	Voltage swell	Voltage dip	THD
	16.32 V	61.81 V	19.14%

## Data Availability

Data are contained within the article.
